# Resilience and adaptation: a mixed-methods exploration of COVID-19’s influence on neonatal residency education in China

**DOI:** 10.1186/s12909-024-05638-1

**Published:** 2024-06-11

**Authors:** Weiqin Liu, Hong Wei, Chunyi Wang, Ziyu Hua

**Affiliations:** 1 Department of Neonatology, National Clinical Research Center for Child Health and Disorders, Ministry of Education Key Laboratory of Child Development and Disorders, Children’s Hospital of Chongqing Medical University, Chongqing, China; 2 Chongqing Key Laboratory of Child Neurodevelopment and Cognitive Disorders, Chongqing, China; 3 Faculty of Internal Medicine, The Pediatric College, Chongqing Medical University, Chongqing, China; 4 National Demonstration Base for Standardized Residency Training, Chongqing, China

**Keywords:** COVID-19, Neonatology residency training, Postgraduate medical education, Mixed-methods approach, Competence

## Abstract

**Background:**

This study aimed to assess the impact of the pandemic of the coronavirus disease 2019 (COVID-19) on neonatology residency training in a tertiary children’s hospital in Chongqing, located in southwest China. Specifically, the study encompassed the effects on residents’ education, lived experiences, well-being, and the quality of neonatal care delivered. As higher educational institutions adapt to the post-COVID-19 era after the pandemic disruption, it is imperative that educational designers/academics learn from their experiences and challenges in curriculum design and delivery, ensuring quality and relevance in education.

**Methods:**

This study employed a mixed-methods approach to investigate the influence of the COVID-19 pandemic on neonatology residency training at a tertiary children’s hospital in Chongqing. The first phase surveyed residents’ perceptions and experiences of their clinical education and well-being during the crisis. The second phase compared the quality of neonatal care between the pre-pandemic and pandemic periods.

**Results:**

The survey of 123 neonatology residents examines the effects of COVID-19 on their learning, training, and mental health. The survey showed that most residents adapted well to the situation. Still, some faced challenges in their clinical education and experiences, such as reduced clinical exposure and opportunities to see rare diseases and conditions. A retrospective analysis of clinical data revealed that 7,151 neonates were admitted to the neonatology department during the study period. There was a 27.6% decrease in neonatal admissions during COVID-19, with more premature births and transfers. Residents conducted fewer clinical procedures but managed more complex cases. During COVID, hospital stays and costs were higher, but antibiotic use was lower. Although the case-mix index (CMI) score increased during the pandemic (1.25 vs. 1.18, *p* < 0.05), there was no significant difference in the rates of readmission within 7 days or poor prognosis.

**Conclusions:**

Despite reduced clinical exposure, the quality of neonatal care was maintained through innovative training methods that enhanced comprehensive residency programs. The study suggested that neonatology residency education remained effective and resilient during the crisis. Exceptional health professional education is vital to train qualified physicians and enhance healthcare systems for future challenges.

**Supplementary Information:**

The online version contains supplementary material available at 10.1186/s12909-024-05638-1.

## Background

The COVID-19 pandemic made a major impact on residency training globally, forcing medical institutions to continually adapt to social distancing measures that have disrupted face-to-face teaching. Many residency programs have had to restructure their curricula and clinical placements rapidly. Some studies have demonstrated that the dearth of clinical placement opportunities is a matter that must be resolved [1–3]. This has prompted many medical and allied healthcare professional programs to implement innovative interventions like online learning and artificial intelligence (AI) [4, 5].

The impact of COVID-19 on medical education has been widely discussed. Still, most of the available information is based on surveys and interviews with residents, susceptible to response bias and institutional variations [6]. Moreover, few studies have objectively compared clinical indicators between the pre-pandemic and pandemic periods, and clinical indicators may not fully capture contextual factors [6]. In addition, little is known about how the pandemic has impacted neonatology residency education, a vital specialty for newborn health. As higher educational institutions adapt to the post-COVID-19 era after the pandemic disruption, it is imperative that educational designers and academics learn from their experiences and challenges in curriculum design and delivery, ensuring quality and relevance in education.

This study aims to fill this gap by using a mixed-methods approach to assess the effects of the pandemic on neonatology residency training in a tertiary children’s hospital in Chongqing, located in southwest China. The first phase will use an online survey to collect residents’ perceptions of their clinical education, experiences, and well-being during the pandemic. The second phase will compare the quality of neonatal care and the graduates’ competencies between pre-pandemic and pandemic periods using a retrospective analysis of clinical data. The study will combine qualitative and quantitative data to assess the effects of COVID-19 on neonatology residents’ education and well-being. Thus, we hope to offer evidence-based recommendations to enhance the quality and effectiveness of neonatology residency training and to prepare healthcare systems for future challenges.

## Methods

### Study design

**Fig. 1:**
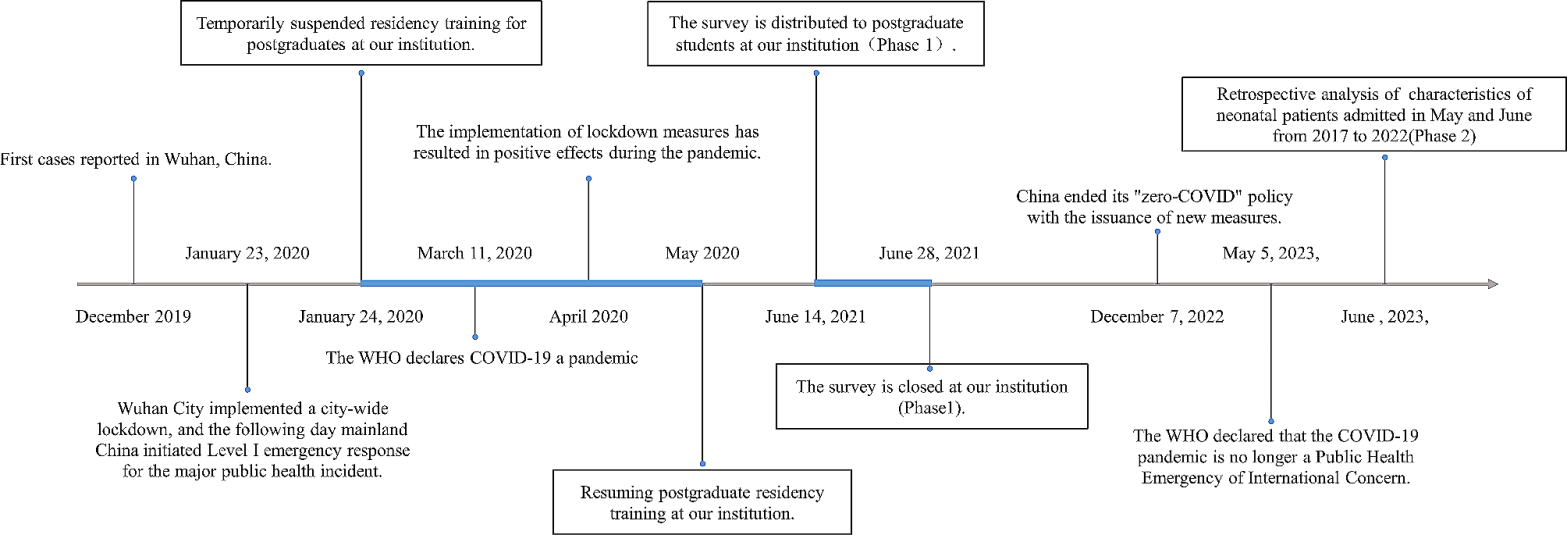
A series of significant pandemic-related events in mainland China and our institution

We used a mixed-methods design to assess the effects of COVID-19 on neonatology residency training in China (shown in Fig. 1).

### Online survey study(phase 1)

To evaluate the impact of COVID-19 on the education and mental health of residents (PGY-2, 3) who completed rotations in our department, we conducted a survey using the “Questionnaire Star” tool from June 14 to June 28, 2021, at a tertiary children’s hospital in Chongqing, southwest China. The web-based survey was distributed to several WeChat working groups of residents, who responded by scanning the Quick Response Code (QR code) provided or by clicking a link. The survey comprised four sections: demographic information, theoretical learning, training cycle, and mental health. The questionnaire was adapted from several recent studies on the effects of COVID-19 on residents [7–11]. This survey was wholly voluntary and non-commercial. The residents who were interviewed were told that the results of the study would be used for scientific research, but they were not informed of the specific research plan. All respondents were anonymous. The questionnaire was exempted from Institutional Review Board (IRB) approval since it was designed to collect only anonymous data.

### Retrospective, observational study(phase 2)

#### Setting

A retrospective observational study was conducted to evaluate the quality of neonatal care during the pandemic. We conducted the study at the Department of Neonatology of Chongqing Medical University’s Children’s Hospital, a tertiary care facility and a referral centre for neonates in southwestern China. The department cares for critically ill newborns and offers a postgraduate residency training program. We obtained ethical approval and waived informed consent from the Children’s Hospital of Chongqing Medical University Human Research Ethics Committee (NO 2,023,284).

#### Participants

The study participants were all patients admitted to our institution’s neonatal unit between May and June 2017 and 2022. Patient demographics and hospital characteristics were retrospectively collected from the electronic medical record (EMR) system. There were no exclusion criteria.

#### Neonatal residency program

**Fig. 2:**
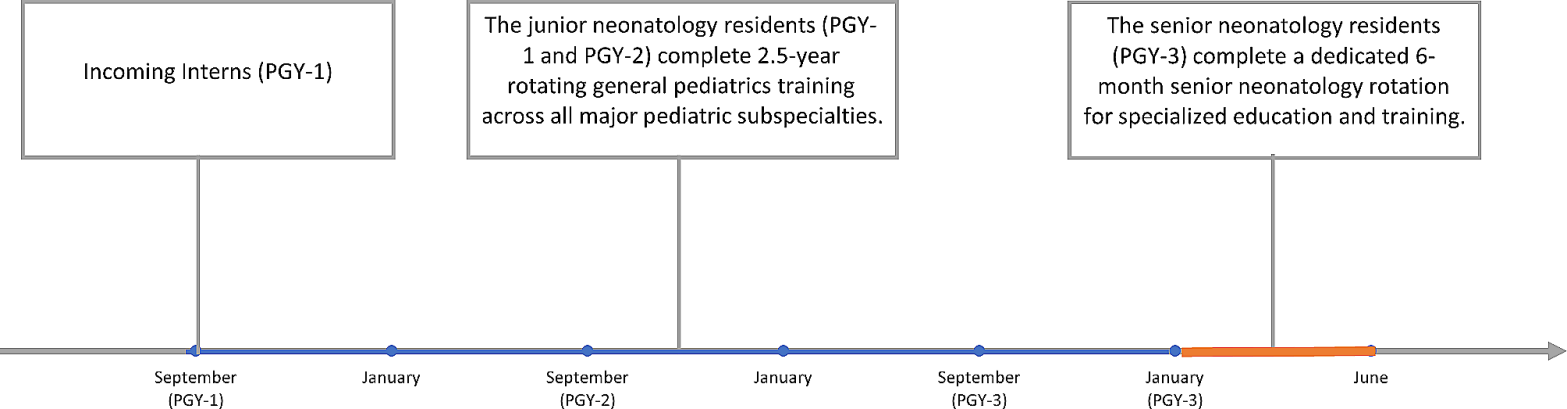
Neonatal postgraduate residency training program in our institution

Our neonatal postgraduate residency training program is a three-year program that prepares postgraduates for working as neonatologists in hospitals. The program consists of two parts: a 2.5-year rotation in various pediatric departments, such as general pediatrics, neonatal ward, pediatric intensive care unit (PICU), pediatric emergency room, community child health, and pediatric outpatient clinics; and a six-month intensive training in the neonatal intensive care unit (NICU), where the Senior Neonatology Resident (Postgraduate Year 3, PGY-3) receives specialized education and clinical experience (shown in Fig. 2).

### Study variables

The following variables were collected to describe the demographic and clinical information of the neonatology clinical cases: admission volume, sex, age (in days), admission weight (in kilograms), gestational age (in weeks + days), admission pathways, therapy, and procedure training, poor prognosis, readmission within 7 days, Case-Mix Index (CMI) scores, length of hospital stay (in days), and hospitalization fees.

The concept of poor prognosis encompassed conditions like death, life-threatening situations, or uncertain outcomes that could benefit from palliative care, including overwhelming sepsis, severe hypoxic-ischemic/neonatal encephalopathy, overwhelming necrotizing enterocolitis totalis, or infants unresponsive to aggressive medical interventions.

The evaluation of resident competency includes but is not limited to, in-service theoretical exams and clinical practice skills assessments.

### Statistics

Descriptive statistics were used to summarize the results of survey items. For continuous variables such as age (in days), birth weight, and hospital length of stay, the median and interquartile range (IQR) were reported. For categorical variables like gestational age groups, gender, and admission pathways, frequencies and percentages were calculated.

Statistical tests were used to compare the characteristics between the pre-COVID and COVID-impacted periods, where appropriate. The Wilcoxon rank-sum and Mann-Whitney U tests were applied to compare continuous variables between groups. Pearson’s chi-squared test was used for comparisons of categorical variables. A *p*-value of less than 0.05 was considered statistically significant. All analyses were performed using R Studio 1.0.44 (RStudio, Inc.) and R v. 4.0.2.

## Results

### Online survey study(phase 1)

#### Impact of COVID-19 pandemic on theoretical learning

Of the 226 individuals who received the questionnaire, 123 completed it, resulting in a response rate of 54.4% (123/226). Most respondents were female (73.4%), with a mean age of 25.9 years (SD 1.0). The postgraduate training levels were 38.2% PGY-2 and 61.8% PGY-3.

Only 26.0% (32/123) of the residents perceived a negative impact of COVID-19 on their theoretical learning. Of this group, only 18.8% (6/32) felt that the effect was moderate to high. Most residents (85.4%, 105/123) used online learning and found it helpful (81.3%, 100/123). However, they also reported negative impacts of the pandemic on theoretical learning, including challenges such as difficulty participating in lengthy online courses without distractions (55.2%), lack of engagement and a conducive learning atmosphere (52.8%), and reduced opportunities for hands-on medical practice (57.7%). (Supplementary Table 1).

#### Impact of COVID-19 pandemic on residency training and well-being

About 48% (59/123) of the residents reported an impact on their training cycle, but only 13.6% (8/59) rated it moderate to high. The main challenges were disrupted training cycles, limited disease spectrum, and reduced hands-on practice. However, the positive impact of the COVID-19 pandemic on residency education includes residents’ improved protective equipment skills and a greater value of life. Most residents (69.9%, 86/123) believed their clinical workload did not change or decrease significantly during the pandemic. We observe a variation in residents’ working hours pre-COVID compared to during the COVID-impacted period. Following the pandemic, there is a notable increase in the proportion of residents working < 40 h (*p* < 0.05), while the proportion working 51–60 h significantly decreased (*p* < 0.05). (shown in Table 1).

**Table 1:** Comparison of weekly residents’ working hours pre-COVID vs. during

Work hours	pre-COVID	COVID impacted	*p* value
< 40 h	3 (2.4)	40 (32.5)	< 0.05
41–50 h	39 (31.7)	29 (23.6)	0.20
51–60 h	47 (38.3)	26 (21.1)	< 0.05
61–70 h	19 (15.4)	16 (13)	0.71
>70 h	15 (12.2)	12 (9.8)	0.68

Most residents (60.2%, 74/123) remained unaffected or optimistic at home. Most residents (82.1%, 101/123) experienced mild or no anxiety about contracting COVID-19 at work. However, 73.2% (90/123) of the residents expressed concern for their families safety during the pandemic (Supplementary Table 2).

### Retrospective, observational study (phase 2)

#### Baseline and demographic characteristics

**Fig. 3:**
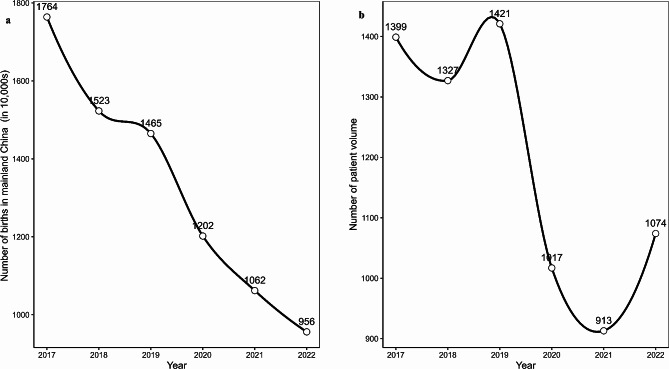
(**a**) Annual Birth Rates in Mainland China from 2017 to 2022 (**b**) Neonatal Admission Trends at Our Institution During the Study Period

A total of 7,151 neonates were admitted to the neonatology department during the study period(Table 2). There was a 27.6% decrease in cases between 2020 and 2022 (the pandemic period) compared to 2017–2019 (the pre-COVID period), from 4,147 to 3,004. The male-to-female ratio was 57.2%. There were no significant differences in postnatal days, gender, or admission weight among the groups. However, there was a shift in admission pathways, characterized by an increase in emergencies and inter-hospital transfers and a decrease in outpatient admissions (*p* < 0.05). During the COVID-impacted period, there was a significant increase in the proportion of premature births, with births occurring at 28 + 0–31 + 6 weeks rising from 5.02 to 6.39% (*p* < 0.05) and a decrease in the proportion of full-term births, with births at ≥ 37 weeks decreasing from 70.6 to 66.9% (*p* < 0.05). The population trends in Mainland China and the institution were analyzed (Fig. 3).

**Table 2:** Baseline characteristics of the study population

Variable		Pre-COVID	COVID impacted	*P* ^1^ value	*P*^2^ value
Total, n		4147	3004		
Age(days), median (IQR)		5.47 [1.28;14.8]	5.63 [1.39;15.4]		0.52
Gestational Age, n (%)	≤ 27^+ 6^wks ≤ 27 + 6	30 (0.72)	36 (1.20)	0.05	< 0.05
	28^+ 0^–31^+ 6^wks 28 + 0–31 + 6	208 (5.02)	192 (6.39)	< 0.05	
	32^+ 0^–36^+ 6^wks 32 + 0–36 + 6	983 (23.7)	766 (25.5)	0.08	
	≥ 37wks ≥ 37	2926 (70.6)	2010(66.9))	< 0.05	
Birthweight(kg) , median (IQR)		3.08 [2.58;3.49]	3.06 [2.53;3.52]		0.48
Gender, Female, n (%)		1751 (42.2)	1308 (43.5)		0.20
Admissions, n (%)	Emergency	204 (4.92)	562 (18.7)	< 0.05	< 0.05
	Outpatient	2227 (53.7)	1049 (34.9)	< 0.05	
	Inter-hospital transport	1716 (41.4)	1393 (46.4)	< 0.05	

Age(days) refers to the age of the neonate at the time of admission

Median (interquartile range, IQR) for continuous variables, number (%) for categorical variables

P2 values represent the statistical comparison between the two groups before and after the COVID-19 pandemic, while P1 values refer to the comparisons within each subgroup across the two time periods

Statistical differences: *p* < 0.05

### Procedural training and neonatal care outcomes and resident competency

The decreased patient volume led to fewer opportunities for therapies and procedures. During the COVID-impacted period, there was a decrease in the proportion of lumbar punctures and endotracheal intubations. However, there was an increase in the proportion of neonates receiving surfactant administration and surgical management. Invasive ventilation decreased, while non-invasive ventilation increased.

The median hospital stay was more extended (8.0 vs. 7.2 days, *p* < 0.05), and the hospital costs were higher (*p* < 0.05) during the COVID-impacted period. The study indicates a significant reduction in antibiotic use during the COVID-19 pandemic, with the DOT/1000 PD value decreasing from 783 before the pandemic to 430 after the pandemic (*p* < 0.05), suggesting a decline in both the intensity and proportion of antibiotic administration among patients. The case-mix index (CMI) score, which reflects the diversity and clinical complexity of the patients, was higher during the COVID-impacted period (1.25 vs. 1.18, *p* < 0.05). The research indicated that re-admission rates within seven days and poor prognosis incidents remained consistent before and during COVID-19. The consistency in in-service theoretical exam scores suggests a stable foundation in theoretical knowledge, while the variation in clinical practice skills assessment scores during the COVID-19 period indicates a subtle shift in practical clinical skills. Its clinical significance is uncertain, warranting further research to ascertain its impact on clinical practice (Table 3).

**Table 3:** Procedural training and neonatal care outcomes and resident competency

Variable		Pre-COVID	COVID impacted	*p* value
Therapeutic procedures	Endotracheal intubation, n (%)	1210 (29.2)	770 (25.6)	< 0.05
	Surfactant administration, n (%)	171 (4.12)	161 (5.36)	< 0.05
	Lumbar puncture, n (%)	697 (16.8)	423 (14.1)	< 0.05
	Surgical management, n (%)	57 (1.37)	107 (3.56)	< 0.05
	Invasive ventilation, n (%)	341 (8.22)	256 (8.52)	0.68
	Non-invasive ventilation, n (%)	506 (12.2)	480 (16.0)	< 0.05
Clinical Outcomes	Antibiotic use (DOT/1000 PD)^a^ median (IQR)	783 [651;847]	430 [416;450]	< 0.05
	Case-Mix Index (CMI) score^b^ (standard deviation)	1.18 (0.05)	1.25 (0.06)	< 0.05
	Length of hospital stay (days) median (IQR)	7.22 [4.91;11.0]	8.02 [5.14;13.1]	< 0.05
	Hospitalization fees (RMB, yuan)^c^ median (IQR)	10,919 [7053;17,592]	11,360 [7676;20,007]	< 0.05
	Readmission within 7 days, n (%)	173 (4.17)	123 (4.09)	0.92
	Poor prognosis, n (%)	56 (1.35)	48 (1.60)	0.45
Exam Scores	In-service theoretical exams^d^	92.0 [90.0;93.0]	92.0 [90.0;95.0]	0.11
	Clinical practice skills assessment^d^	90.0 [88.0;91.0]	89.0 [88.0;90.0]	< 0.05

a DOT/1000 PD days of therapy per 1000 patient days. DOT is calculated by multiplying the number of doses received by the dosing interval and then dividing by 24 h for each antibiotic the patient receives, and PD is calculated by multiplying the total number of admissions with the average length of stay

b CMI: The case mix index is calculated by adding up the relative Medicare Severity Diagnosis Related Group (MS-DRG) weight for each discharge and dividing that by the total number of Medicare and Medicaid discharges in a given month and year

c RMB: renminbi 1 dollar ≈ 7 .2RMB (2023)

d The exam is graded on a scale of 100 points

Median (interquartile range, IQR) for continuous variables, number (%) for categorical variables

Statistical differences: *p* < 0.05

## Discussion

We conducted a mixed-methods study to examine how the COVID-19 pandemic affected the quality and delivery of neonatology resident education in a tertiary care hospital in China. We found that the pandemic disrupted traditional teaching and learning modes and reduced the residents’ clinical exposure and skill development. However, we also found some positive outcomes, such as the adoption of online platforms and the emergence of new competencies related to COVID-19 prevention and control. Utilizing multiple indicators such as stable readmission rates, decreased antibiotic utilization, increased non-invasive ventilation, and unchanged poor prognosis rates, the study demonstrates that despite disruptions from the pandemic, the quality of neonatal care and residency education remained robust through adaptations and innovative training approaches.

For a long time, traditional bedside teaching on rounds has been considered the “heart and soul” of training. The COVID-19 pandemic disrupted clinical placements for students worldwide. Medical institutions, such as online simulations and virtual patients, adopted remote and multi-modal strategies to cope with this challenge [5, 12, 13]. These remote and multi-modal educational approaches offer both advantages and disadvantages. The advantages include providing a safe virtual practice environment and increasing flexibility [14]. Our survey of neonatal residents indicated that online learning was widely embraced and perceived as beneficial, with minimal impact on theoretical knowledge acquisition during the pandemic. However, they also have limitations, such as an inability to fully cultivate essential skills like communication, empathy, and bedside manner, and a lack of hands-on practice that may hinder the acquisition of procedural skills. Moreover, their effectiveness can be influenced by technological factors [15, 16]. These remote and multi-modal educational approaches are still in progress. Still, they are likely to persist, posing new challenges and opportunities to improve teaching and learning in the health professions [5].

The travel restrictions imposed during the pandemic significantly curtailed traditional bedside teaching. This limited residents’ opportunities to perform comprehensive physical examinations, potentially leading them to miss rare conditions or overlook unique cases crucial for learning and developing clinical experience. Furthermore, the shift away from the bedside eliminated real-time workroom teaching and discussion, a valuable component of traditional rounds. While the training period for traditional bedside teaching was shortened, it’s important to note that learning was not entirely halted but rather took on different formats, such as remote and multi-modal educational approaches. The implications and effectiveness of these alternate training methods necessitate long-term follow-ups and observations of the residents. Our study also highlights these otential drawbacks of the curtailed training periods but did not extend its exploration to cover the implications of this on graduation. Other research has raised concerns from residents and programme directors about shortened training periods adversely affecting board exam pass rates. Extending programmes and delaying board exams were considered potential solutions, though such measures could impact residents’ career plans and quality of life [17, 18].

Following relaxed travel limits, residents gradually resumed clinical work but faced challenges: a significant drop in attendance and workload and a shift in working hours during the pandemic. Several studies revealed that workload changes reduced clinical exposure, particularly involving non-critical patients and elective surgeries, hindering residents’ skill development and preparedness for future careers [3, 6, 19]. The impact of COVID-19 on resident working hours varied across hospitals and specialties. Our survey data aligns with some studies, showing a decrease in specific resident work hour categories during COVID-19, likely due to reduced patient volume [20, 21]. However, some studies documented increased resident burdens due to COVID-related work [6]. While a statistically significant difference was present in clinical practice scores during the pandemic, the modest score variation does not necessarily suggest substantial competency gaps. As an academic medical center, our experienced faculty of neonatologists likely mitigated any potential impact of these gaps through close supervision and mentorship. Furthermore, the clinical practice skills assessment is a subjective evaluation that may not fully capture residents’ actual clinical competencies, which are honed through continuous practice and feedback from supervising faculty. So, overemphasising minor subjective score changes could divert attention from the successful adaptation and resilience demonstrated by the residency program.

Moreover, Travel restrictions increased the rate of preterm and surgically managed infants and the CMI for inpatients admitted during the pandemic in our institution. This provided residents with the opportunity to learn from a higher proportion of complex or critically ill neonates. The increase in the number of critically ill patients admitted during the pandemic is consistent with the findings of other studies [22]. However, consultations and non-critical cases were lower during the pandemic. Studying non-critical illnesses is essential for building a foundation for effective patient care and gaining skills. Ensuring that residents balance critical and non-critical conditions is important for medical education and patient care. Admitting a higher proportion of complex or critically ill neonates may increase the likelihood of errors by residents, prolong patient stays, and raise costs. However, our data indicated no change in prognosis and re-admissions within seven days between the two periods (*p*>0.05). This suggested that neonatology residents’ quality of care and education remained unaffected.

The pandemic challenged the psychological wellness of residents but also highlighted the role of health professional education in helping them cope [5]. Our survey found that most residents remained hopeful and managed their stress. Health-professional education can teach them skills to balance their time, work, and life and to find meaning and purpose in their work. These skills are part of “learning to live one’s own life,” a key element of the “education for life” framework. This framework supports lifelong learning and well-being competencies enabled by new environments or challenges. Even in hard times, residents can find meaning and purpose by learning to live their own lives [23, 24]. In addition, the pandemic offered unique learning opportunities for residents. They learned about pandemic management, such as using personal protective equipment, triaging patients, preventing infection spread, and ventilator and antibiotic optimization. These skills stimulated residents to adjust and augment their abilities and confidence.

As the COVID-19 pandemic wanes, it is expected that patient volumes in most medical specialties will gradually return to their pre-pandemic levels. However, neonatal specialties may still face the challenge of limited clinical exposure due to China’s persistently low fertility rate [25–27]. To address this, innovative educational approaches, such as simulation and AI, are crucial for providing comprehensive training. These approaches, including virtual patients, mannequins, serious games, and augmented reality, offer safe and authentic learning experiences across various patient-care settings [28, 29]. They enable residents to acquire the essential skills for effective neonatal care. Trainees are expected to identify and address knowledge gaps through self-directed learning. Lifelong learning is vital, rather than fixed stages of education, work, and retirement. Open educational systems should replace closed ones tailored to the evolving needs of healthcare professionals throughout their careers. This approach allows for flexibility and adaptability in education, ensuring that professionals can update their skills and knowledge in response to the changing healthcare landscape [5].

Our study has several limitations. First, it was based on a single institution, postgraduate students, and medical data from May and June, which may not represent the neonatal training quality across different settings, region and periods. Second, we excluded some relevant questions from the questionnaire, particularly standardized depression scales such as the Patient Health Questionnaire-9 (PHQ-9) or the Centre for Epidemiological Studies Depression Scale (CES-D), for brevity, comfort, and ethics [30, 31]. However, this compromised the questionnaire’s reliability, validity, and bias. Thirdly, the pandemic has also influenced the employment of residents. Further studies, such as surveys, are necessary to investigate how the pandemic has reshaped the demand and supply of residents, as well as the economic implications of these changes for policy and practice. Finally, we did not explore attending physicians’ competency or residents’ leadership characteristics during the pandemic in our study. Our research concentrated on the pandemic’s impact on resident training and clinical experiences. These aspects are crucial for patient care and medical education and warrant further investigation in future research.

## Conclusion

The COVID-19 pandemic significantly affected the traditional modes of teaching and learning and limited the clinical exposure and skill development of neonatology residents in our institution. However, the pandemic also prompted the adoption of online platforms and the emergence of new skills related to COVID-19 prevention and control. The quality of care and education remained stable and resilient during the crisis through adaptation and innovation. Exceptional health professional education is vital to train qualified physicians and enhance healthcare systems for future challenges.

### Electronic supplementary material

Below is the link to the electronic supplementary material.

Supplementary Material 1

Supplementary Material 2

## Data Availability

The datasets used and/or analysed during the current study are available from the corresponding author on reasonable request.

## Abbreviations

CMI: Case mix index
COVID-19: Coronavirus disease 2019
EMR: Electronic medical record
IQR: Interquartile range
IRB: Institutional review board
NICU: Neonatal intensive care unit
PGY-3: Postgraduate Year 3
PICU: Pediatric intensive care unit
RMB: Renminbi
