# Supplementary material for: Resilience and adaptation: a mixed-methods exploration of COVID-19’s influence on neonatal residency education in China
Source: BMC Med Educ. 2024 Jun 11;24:654. doi: 10.1186/s12909-024-05638-1 (PMC11167867; doi:10.1186/s12909-024-05638-1)
Supplement: Supplementary file 1 — Supplementary Material 1 [file 12909_2024_5638_MOESM1_ESM.doc]

**Supplementary Tab. 1 Participants’ Demographics and COVID-19’s Impact on Theoretical Learning**

| **Characteristic** | Number of Respondents (n) | Percent of Respondents (%) |
| --- | --- | --- |
| **Demographic information** |  |  |
| Overall | 123 | 100 |
| **1. Gender** |  |  |
| Male | 46 | 26.6 |
| Female | 127 | 73.4 |
| Not answered | 0 | 0 |
| **2.Mean Age(SD)** | 25.9 ( 1.0) |  |
| **3. Ethnicity** |  |  |
| Han Chinese | 114 | 92.7 |
| Ethnic Minority | 9 | 7.3 |
| **4. Marital status** |  |  |
| Single | 89 | 72.4 |
| Married | 11 | 8.9 |
| Not answered | 23 | 18.7 |
| **5. Years of Postgraduate Training** |  |  |
| PGY-2 (Postgraduate Year 2) | 47 | 38.2 |
| PGY-3 (Postgraduate Year 3) | 76 | 61.8 |
| **COVID-19 disrupts theoretical learning** |  |  |
| **6. Has the COVID-19 pandemic impacted clinical educational activity?** |  |  |
| Yes | 32 | 26.0 |
| NO | 77 | 62.6 |
| Uncertainty | 14 | 11.4 |
| **7. If yes, to what extent do you think it has influenced theoretical learning?** |  |  |
| Very low influence (＜20%) | 8 | 25.0 |
| Low influence (21-40%) | 18 | 56.3 |
| Moderate influence (41-60%) | 5 | 15.6 |
| High influence (61-80%) | 1 | 3.1 |
| Very high influence (＞80%) | 0 | 0 |
| **8. Access to clinical educational activity during the COVID-19 pandemic? （choose any that apply）** |  |  |
| Online courses | 105 | 85.4 |
| WeChat | 66 | 53.7 |
| Web browsing | 87 | 70.7 |
| Online meeting | 78 | 63.4 |
| Teaching rounds | 53 | 43.1 |
| Other | 52 | 42.3 |
| **9.** **Do you think online courses are helpful for theoretical learning in training?** |  |  |
| Yes | 100 | 81.3 |
| No | 0 | 0 |
| Uncertainty | 23 | 18.7 |
| **10. The negative impacts of the pandemic on theoretical learning in training（choose any that apply）** |  |  |
| Interaction between students and teachers is limited, and doubts cannot be resolved in time | 62 | 50.4 |
| It is challenging to participate in a lengthy online course without being distracted. | 68 | 55.2 |
| Lack of "engagement" in the online course and a learning atmosphere. | 65 | 52.8 |
| Most of the time, it focuses on COVID-19 and less on other topics. | 22 | 17.9 |
| Reduced opportunities for hands-on medical practice. | 71 | 57.7 |
| Other | 17 | 13.8 |

SD： the standard deviation
