# Supplementary material for: Resilience and adaptation: a mixed-methods exploration of COVID-19’s influence on neonatal residency education in China
Source: BMC Med Educ. 2024 Jun 11;24:654. doi: 10.1186/s12909-024-05638-1 (PMC11167867; doi:10.1186/s12909-024-05638-1)
Supplement: Supplementary file 2 — Supplementary Material 2 [file 12909_2024_5638_MOESM2_ESM.doc]

**Supplementary Tab. 2 COVID-19’s Impact on Residency Training and Mental Health.**

| **Characteristic** | Number of Respondents (n) | Percent of Respondents (%) |
| --- | --- | --- |
| **COVID-19 Disrupts Residency Training Cycle** |  |  |
| **1. Do you think the COVID-19 pandemic has affected the training cycle in residency education** |  |  |
| Yes | 59 | 48.0 |
| NO | 48 | 39.0 |
| Uncertainty | 16 | 13 |
| **2. If yes, to what extent do you think it has affected the planning of the training cycle in residency education?** |  |  |
| Very low influence (＜20%) | 26 | 44,1 |
| Low influence (21-40%) | 25 | 42.4 |
| Moderate influence (41-60%) | 7 | 11.9 |
| High influence (61-80%) | 1 | 1.6 |
| Very high influence (＞80%) | 0 | 0 |
| **3.The negative impact of the COVID-19 pandemic on the training cycle in residency education includes:（choose any that apply）** |  |  |
| Decrease or absence of hands-on practice | 62 | 50.4 |
| Some increase in workload due to staffing constraints | 57 | 46.3 |
| Some departments cancel or shorten residency training cycles. | 90 | 73.2 |
| Missing or reduced partial disease spectrum, resulting in a lack of corresponding clinical experience | 67 | 54.5 |
| Other | 11 | 8.9 |
| **4.The positive impact of the COVID-19 pandemic on the training cycle in residency education includes（choose any that apply）** |  |  |
| Became proficient in the operation of various personal protective equipment and learned more about infectious diseases | 95 | 77.2 |
| Increased awareness of respiratory-related diseases | 73 | 59.3 |
| Although the clinical workload has decreased, it has given me more time to get to know my patients | 21 | 17 |
| There was more time to interact and learn from senior doctors | 20 | 16.3 |
| The pandemic has partially influenced my outlook on life, with a greater reverence for life and a greater love of life | 80 | 65.0 |
| Other | 12 | 9.8 |
| **5. Working hours per week before the COVID-19 pandemic** |  |  |
| <40 hours | 3 | 2.4 |
| 41-50 hours | 39 | 31.7 |
| 51-60 hours | 47 | 38.3 |
| 61-70 hours | 19 | 15.4 |
| ＞70 hours | 15 | 12.2 |
| **6. Working hours per week during the COVID-19 pandemic** |  |  |
| <40 hours | 40 | 32.5 |
| 41-50 hours | 29 | 23.6 |
| 51-60 hours | 26 | 21.1 |
| 61-70 hours | 16 | 13.0 |
| ＞70 hours | 12 | 9.8 |
| **7. Has there been a change in clinical workload during the pandemic?** |  |  |
| Increased significantly | 37 | 30.1 |
| No significant change | 44 | 35.8 |
| Decreased significantly | 42 | 34.1 |
| **COVID-19 impacts resident mental health** |  |  |
| **8. As a resident, what was your general state of mind at home during the COVID-19 pandemic?** |  |  |
| Happy | 22 | 17.9 |
| Depressed | 6 | 4.0 |
| Anxious | 24 | 19.5 |
| No difference, same as usual | 52 | 42.3 |
| Uncertain | 19 | 16.3 |
| **9. How stressed and/or anxious did you feel about contracting COVID-19 during the COVID-19 pandemic?** |  |  |
| Extremely stressed or anxious | 4 | 3.3 |
| Moderately stressed or anxious | 16 | 13.0 |
| Mildly stressed or anxious | 81 | 65.8 |
| No difference, same as usual | 20 | 16.3 |
| Uncertain | 2 | 1.6 |
| **10. Did you express concern for family safety during the COVID-19 pandemic?** |  |  |
| Yes | 90 | 73.2 |
| NO | 24 | 19.5 |
| Uncertain | 9 | 7.3 |
